# Alterations in the muscle-to-capillary interface in patients with different degrees of chronic obstructive pulmonary disease

**DOI:** 10.1186/1465-9921-11-97

**Published:** 2010-07-15

**Authors:** Gabriella Eliason, Samy M Abdel-Halim, Karin Piehl-Aulin, Fawzi Kadi

**Affiliations:** 1 School of Medical Sciences, Örebro University, Örebro, Sweden; 2 Department of Medical Sciences, Respiratory Medicine and Allergology, Uppsala University, Uppsala, Sweden; 3 Department of Rheumatology, Danderyds hospital, Stockholm, Sweden

## Abstract

**Background:**

It is hypothesized that decreased capillarization of limb skeletal muscle is implicated in the decreased exercise tolerance in COPD patients. We have recently demonstrated decreased number of capillaries per muscle fibre (CAF) but no changes in CAF in relation to fibre area (CAFA), which is based on the diffusion distance between the capillary and muscle fibre. The aim of the current study is to investigate the muscle-to-capillary interface which is an important factor involved in oxygen supply to the muscle that has previously been suggested to be a more sensitive marker for changes in the capillary bed compared to CAF and CAFA.

**Methods:**

23 COPD patients and 12 age-matched healthy subjects participated in the study. Muscle-to-capillary interface was assessed in muscle biopsies from the tibialis anterior muscle using the following parameters:

1) The capillary-to-fibre ratio (C:F_i_) which is defined as the sum of the fractional contributions of all capillary contacts around the fibre

2) The ratio between C:F_i _and the fibre perimeter (CFPE-index)

3) The ratio between length of capillary and fibre perimeter (LC/PF) which is also referred to as the index of tortuosity.

Exercise capacity was determined using the 6-min walking test.

**Results:**

A positive correlation was found between CFPE-index and ascending disease severity with CFPE-index for type I fibres being significantly lower in patients with moderate and severe COPD. Furthermore, a positive correlation was observed between exercise capacity and CFPE-index for both type I and type IIa fibres.

**Conclusion:**

It can be concluded that the muscle-to-capillary interface is disturbed in the tibialis anterior muscle in patients with COPD and that interface is strongly correlated to increased disease severity and to decreased exercise capacity in this patient group.

## Introduction

COPD (chronic obstructive pulmonary disease) is a disease characterized by irreversible airflow obstruction [1]. A significant number of patients with COPD develop skeletal muscle wasting and decreased exercise capacity [2-5]. Previous studies have demonstrated the occurrence of a shift towards fatigue-susceptible anaerobic glycolytic muscle properties relative to the aerobic oxidative muscle properties [6-9]. We have previously shown that changes in fibre type composition occur in the later stages of COPD while exercise capacity is decreased already in mild and moderate COPD, indicating that other factors also may influence decreased exercise capacity in these patients [10]. Exercise tolerance is partly dependent on the oxidative capacity of the skeletal muscle and an important limiting factor for exercise capacity in COPD is the oxygen supply to the muscle [11]. The oxidative metabolism in skeletal muscle is dependent on the mitochondrial volume density and activity and on the capillary supply. Therefore alterations in muscle capillary network or mitochondria can cause decreased exercise tolerance in COPD. Indeed, a previous study has reported lower mitochondrial volume density but no changes in mitochondrial respiratory function in patients with COPD [12]. Furthermore, three previous studies have suggested a decreased number of capillaries/muscle fibre (CAF) in patients with COPD [8,9,13]. However, it is important to highlight the fact that the ratio between the number of capillaries and the area of muscle fibres (CAFA) is not significantly altered in COPD patients [8,9,13]. This finding can be explained by a reduction in fibre area as previously shown in COPD patients [10]. This is also in line with a previous study [9] indicating a parallel reduction in the number of capillaries around the fibre and the area of the muscle fibre in COPD patients. Therefore based on the capillary parameter CAFA, muscle capillarization is not decreased in COPD patients.

The capillary supply is usually assessed by counting the number of capillaries around each fibre (CAF) or by computing the ratio between CAF and the area of the muscle fibre (CAFA). CAFA is a parameter based on the diffusion distance between the capillary and the centre of the fibre. Capillary parameters essentially determining the diffusion distance may not detect actual disturbances in muscle capillarization. It has previously been suggested that the muscle-to-capillary interface is an important factor involved in oxygen supply to the muscle [14-19] and may thereby be used as a more sensitive marker for changes in the capillary bed compared to CAF and CAFA [15,16]. To asses muscle-to-capillary interface precise stereological procedures such as the capillary-to-fibre perimeter ratio have been used [20]. Some stereological methods cannot be used in human studies since muscle samples need to be perfused and fixed in order not to collapse. However, capillary-to-fibre ratio for an individual fibre (C:F_i_) can be assessed by determining the number of capillaries around the fibre and the sharing factor (SF) for each fibre and thereafter calculating the sum of the fractional contributions of each capillary contact. Thereafter the capillary-to-fibre perimeter exchange index (CFPE-index) can be calculated as the quotient C:F_i _and fibre perimeter [19]. As CFPE-index has been shown to be correlated to precise stereological methods it can be used to assess muscle fibre-to-capillary interface in human studies [15,19].

Measuring the percentage of fibre perimeter in contact with the capillary wall (index of tortuosity (LC/PF)) which is based on the length of capillaries, the number of capillaries and the perimeter of the fibre is another sensitive method for assessment of muscle-to-capillary interface which also takes in account the capillary geometry [21]. To our knowledge CFPE-index and LC/PF have not been evaluated in COPD and the question of whether muscle-to-capillary interface is altered in this patient group remains unknown.

Given earlier reports suggesting disturbed limb skeletal muscle capillarization in COPD patients [8,13] the current study aim was to examine the muscle-to-capillary interface in different stages of COPD and its correlation with the degree of airflow obstruction and exercise capacity.

## Materials and methods

### Study population

Twenty-three COPD patients (10 males and 13 females) mean age 62.0 ± 6.6 years, were recruited from the Department of Respiratory Medicine at Örebro University Hospital (Table 1). The patients were selected in a stable condition and were not suffering from any respiratory tract infections or exacerbations of their disease four weeks prior to sampling date. Exclusion criteria were malignancy, cardiac failure and severe endocrine-, hepatic- or renal disorder. Based on the severity of airflow obstruction the patients were divided into three subgroups based on the "Global Initiative for Chronic Obstructive Lung Disease (GOLD)" criteria [1]. Eight patients (four males and four females) had mild COPD (forced expiratory volume in 1 s (FEV_1.0 _) > 80% of predicted), nine patients (two males and seven females) had moderate COPD (FEV_1.0 _30-80% of predicted) and six patients (four males and two females) had severe COPD (FEV_1.0 _< 30% of predicted). Twelve age-matched, healthy, non-smoking subjects (n = 12, 6 male, 6 female) were recruited as a control group (Table 1).

**Table 1:** Anthropometry and exercise capacity in 23 COPD patients and 12 age-matched healthy subjects.

	*Healthy* *Subjects* *(n = 12)*	*Mild COPD (n = 8)*	*Moderate COPD* *(n = 9)*	*Severe* *COPD* *(n = 6)*	*P-value*
Age (years)	61.9 ± 7.9	60.8 ± 7.5	61.1 ± 3.8	64.0 ± 8.7	ns
Height (cm)	170.2 ± 10.4	169.5 ± 10.4	170.5 ± 8.2	167.7 ± 6.3	ns
Weight (kg)	77.1 ± 12.4	78.4 ± 21.0	82.4 ± 22.0	66.4 ± 11.3	ns
BMI (kg/m^2^)	26.6 ± 3.6	27.0 ± 5.4	28.0 ± 5.5	23.6 ± 3.9	ns
FEV_1.0 _(% of expected)	115 ± 12	86 ± 6	48 ± 8*	26 ± 3*	< 0.001
PaO_2 _(kPa)	11.1 ± 1.3	10.5 ± 1.5	9.5 ± 1.1	9.2 ± 1.1*	0,01
PaCO_2 _(kPa)	5.2 ± 0.3	4.8 ± 0.3	5.0 ± 0.6	5.6 ± 0.7*	0.02
Distance walked in 6 min (m)	502 ± 52	422 ± 43	336 ± 46*	248 ± 91*	< 0.001

Data are presented as mean ± SD.

* Significant difference compared to healthy subjects.

n = number of test subjects; BMI = body mass index; FEV_1.0 _= forced expiratory volume in one second; PaO2 = partial arterial pressure for oxygen; PaCO2 = partial arterial pressure for carbon dioxide; ns = not significant.

Written informed consent was obtained from all subjects before their participation in the study, which was approved by the Ethics Board of Uppsala University, Sweden (dnr 2004:M-355).

### Pulmonary function tests

All patients and age-matched healthy subjects underwent a spirometry with reversibility test to determine FEV_1.0 _with the highest value from at least three technically acceptable assessments being used.

### Blood samples

All participants were sampled for arterial blood gases from the radial artery at rest. The samples were analysed for partial arterial pressure for oxygen (PaO_2_) and carbon dioxide (PaCO_2_).

### Exercise capacity test

Exercise capacity was determined using a 6 min walking test performed on a 25 meter "court" as previously reported [10] (Table 1).

### Muscle samples

Muscle biopsies were obtained from the bulk of the tibialis anterior muscle, which is an important postural muscle active daily for long periods and involved in balance control and foot stability during walking [22], under local anaesthesia (Xylocaine^®^2%) as previously described [10,13,23]. The biopsies were frozen in isopentane cooled to its freezing point in liquid nitrogen and stored in -80°C until analyses were performed.

### Immunohistochemistry

Serial transverse sections, 5 μm thick, were cut at -22°C using a microtome (Leica CM1850, Leica Microsystems, Germany) and mounted on glass slides. Muscle fibre composition was determined by immunohistochemical staining using the monoclonal antibodies N2.261 and A4.951 (Developmental Studies Hybridoma Bank, University of Iowa) [24] as previously described [10,13] (Table 2). Fibres of type I, type IIa, type IIx, type IIx-a and type IIa-b were determined. Fibre area and fibre perimeter for type I and type IIa fibres were determined on four to ten randomly selected areas (table 2). For the visualization of capillaries the monoclonal antibody CD31 (Dako, Glostrup, Denmark; MO823) was used [13,16]. For visualization of the fibre cytoplasm the histological staining using eosin was applied. CD 31 has been used for the identification of capillaries in several studies and a comparison between CD 31 staining and α-amylase-PAS for identifying capillaries showed that the use of both methods results in a similar number of capillaries counted by the observer and that CD31 staining allows an easier visualization of capillaries [16]. Sequential estimation analyses indicate that 50 fibres from one biopsy are sufficient to characterise capillary parameters [25]. In the present study capillaries in contact with oxidative type I fibres and glycolytic type IIa fibres were analysed from pictures taken with a magnitude of ×20 obtained from four to ten randomly selected cross-sectional areas corresponding to a mean of 80 fibres from each biopsy.

**Table 2:** Fibre type distribution, fibre area, fibre perimeter, CAF and CAFA for type I and type IIa fibres.

	Healthy subjects (n = 12)	Mild COPD (n = 8)	Moderate COPD (n = 9)	Severe COPD (n = 6)	P-value
Proportion type I fibres (%)	78.3 ± 8.7	70.2 ± 11.4	74.5 ± 11.9	59.4 ± 9.5*	0.009
Proportion type IIa fibres (%)	20.2 ± 8.9	24.6 ± 10.6	22.6 ± 11.4	40.2 ± 7.6*	0.02
Proportion type IIx fibres (%)	0	1.0 ± 1.6	0.6 ± 1.1	1.2 ± 2.1	ns
Proportion type IIx-a fibres (%)	0	0.2 ± 0.3	0.3 ± 0.6	0.5 ± 0.7	ns
Proportion type I-IIa fibres (%)	1.5 ± 1.4	4.0 ± 6.4	2.0 ± 2.3	1.6 ± 1.4	ns
Perimeter type I fibres	330 ± 38	332 ± 60	295 ± 40	320 ± 87	ns
Perimeter type IIa fibres	380 ± 48	348 ± 56	274 ± 53*	303 ± 81	0,003
Area type I fibres (μm^2^)	7143 ± 1508	7315 ± 2471	5736 ± 1497	6557 ± 2901	ns
Area type IIa fibres (μm^2^)	9262 ± 2215	7711 ± 2427	4880 ± 1970*	5418 ± 2232	0,004
CAF type I fibres (μm)	6,8 ± 1,1	6,8 ± 2,6	5,2 ± 0,9*	5,1 ± 1,2*	0,006
CAF type IIa fibres (μm)	6,7 ± 1,9	6,3 ± 2,4	4,4 ± 0,8*	4,5 ± 1,0*	0,002
CAFA type I fibres	1,0 ± 0,1	1,1 ± 0,4	1,0 ± 0,3	0,9 ± 0,2	ns
CAFA type IIa fibres	0,8 ± 0,3	1,0 ± 0,3	1,1 ± 0,3	1,0 ± 0,3	ns

Data are presented as mean ± SD.

*Significant difference compared to healthy subjects.

n = number of test subjects; CAF = number of capillaries around a single fibre; CAFA = the ratio between CAF and the area of the muscle fibre; ns = not significant

In a previous study we have reported the number of capillaries around a single muscle fibre (CAF) and the ratio between CAF and the fibre area (CAFA) [13] (table 2). The capillary parameters measured in transverse sections of the muscle biopsies in the present study were:

1) The capillary-to-fibre ratio (C:F_i_), which was calculated by determining the number of capillaries around for the fibre in question followed by determination of the sharing factor (SF) for each capillary and thereafter taking the sum of the fractional contributions of all capillary contacts around the fibre [19].

2) The quotient between C:F_i _and the fibre perimeter, i.e. the CFPE-index [19]

3) The ratio between length of capillary and fibre perimeter (LC/PF) which represents the percent of muscle fibre perimeter in contact with capillary wall. LC/PF is also referred to as the index of tortuosity [15,16].

### Statistic analysis

Statistics were performed using Statistix^®^8 (Analytic Software, Tallahassee).

All data are presented as mean ± standard deviation. For comparison between groups the Kruskal-Wallis one way ANOVA test was used. When significance was found the Kruskal-Wallis all pairwise comparison post-hoc test was applied. Relationships between variables were studied using Spearmans rank correlation test. p < 0.05 was considered to be significant.

## Results

The following parameters associated with muscle-to-capillary interface were assessed in the COPD population: the capillary-to-fibre ratio (C:F_i_), the capillary-to-fibre perimeter exchange index (CFPE-index) and the index of tortuosity (LC/PF).

The C:F_i _for type I fibres was significantly lower (p = 0.007) in the groups with moderate and severe COPD compared with the age-matched healthy subjects and the C:F_i _for type IIa fibres was significantly lower (p = 0.002) in the group with severe COPD compared to the age-matched healthy subjects, indicating that each capillary is shared by more muscle fibres in these patient groups (Table 3). We also found that CFPE-index for type I fibres was significantly lower (p = 0.002) in the groups with moderate and severe COPD compared with the age-matched healthy subjects (Table 3).

**Table 3:** Muscle-capillary interface parameters for type I and type IIa fibres.

	*Healthy* *subjects* *(n = 12)*	*Mild* *COPD* *(n = 8)*	*Moderate* *COPD* *(n = 9)*	*Severe* *COPD* *(n = 6)*	*p value*
C:F_i _type I	2.7 ± 0.5	2.7 ± 1.3	2.0 ± 0.4*	2.0 ± 0.5*	0.007
C:F_i _type IIa	2.5 ± 0.5	2.5 ± 1.1	1.6 ± 0.4*	1.7 ± 0.4	0.002
CFPE type I	8.3 ± 1.1	8.1 ± 3.0	6.8 ± 0.9*	6.2 ± 0.6 *	0.002
CFPE type IIa	6.6 ± 0.9	7.3 ± 2.6	5.9 ± 0.8	5.8 ± 1.1	ns
LC type I	72.4 ± 8.4	77.0 ± 25.0	58.8 ± 14.6	67.4 ± 29.0	ns
LC type IIa	64.8 ± 19.5	64.7 ± 17.8	45.2 ± 10.6	57.4 ± 25.8*	0.03
LC/PF type I	21.8 ± 1.4	22.3 ± 5.6	20.0 ± 4.7	20.4 ±4.1	ns
LC/PF type IIa	17.5 ± 2.5	19.0 ± 4.1	16.3 ± 2.2	18.2 ± 2.5	ns

Data are presented as mean ± SD.

*Significantly lower compared to healthy subjects.

n = number of test subjects; C:F_i _= the sum of the fractional contributions of all capillary contacts around the fibre, i.e. the individual capillary-to-fibre ratio; CFPE = quotient between C:F_i _and the fibre perimeter; LC = length of capillaries in contact with muscle fibre; LC/PF = ratio between LC and fibre perimeter; ns = not significant.

There were no significant differences in LC/PF between the different groups (Table 3). However, the length of capillaries in contact with type IIa fibres (LC type IIa) was significantly lower (p = 0.03) in the group with moderate COPD compared to healthy subjects.

**Figure 1:**
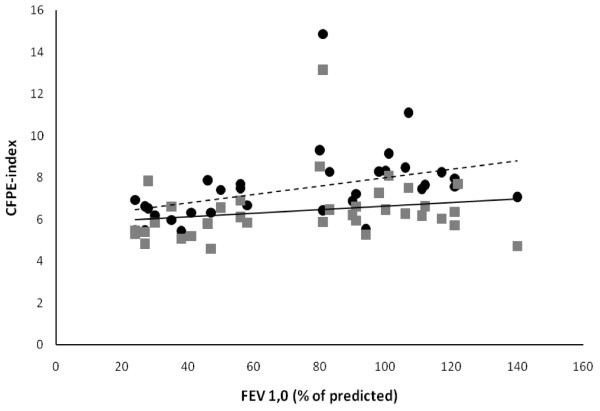
**Relationship between degree of airflow obstruction expressed as percent of predicted FEV_1.0 _and CFPE-index for type I and type IIa fibres; "black circle" = type I fibres (--- = regression line for type CFPE-index for type I fibres, r = 0.61, p < 0.001), "grey square"= type IIa fibres (-- = regression line for CFPE-index for type IIa fibres, r = 0.37, p = 0.04); FEV_1,0 _= forced expiratory volume in one second; CFPE-index = quotient between individual capillary-to-fibre ratio and fibre perimeter**.

A positive correlation was seen between the degree of airflow obstruction expressed as percent of predicted FEV_1.0 _and CFPE-index for both type I fibres (r = 0.61, p < 0.001) and type IIa fibres (r = 0.37, p = 0.04) (Fig 1) likely indicating that each capillary is shared by more muscle fibres when airflow obstruction increases.

**Figure 2:**
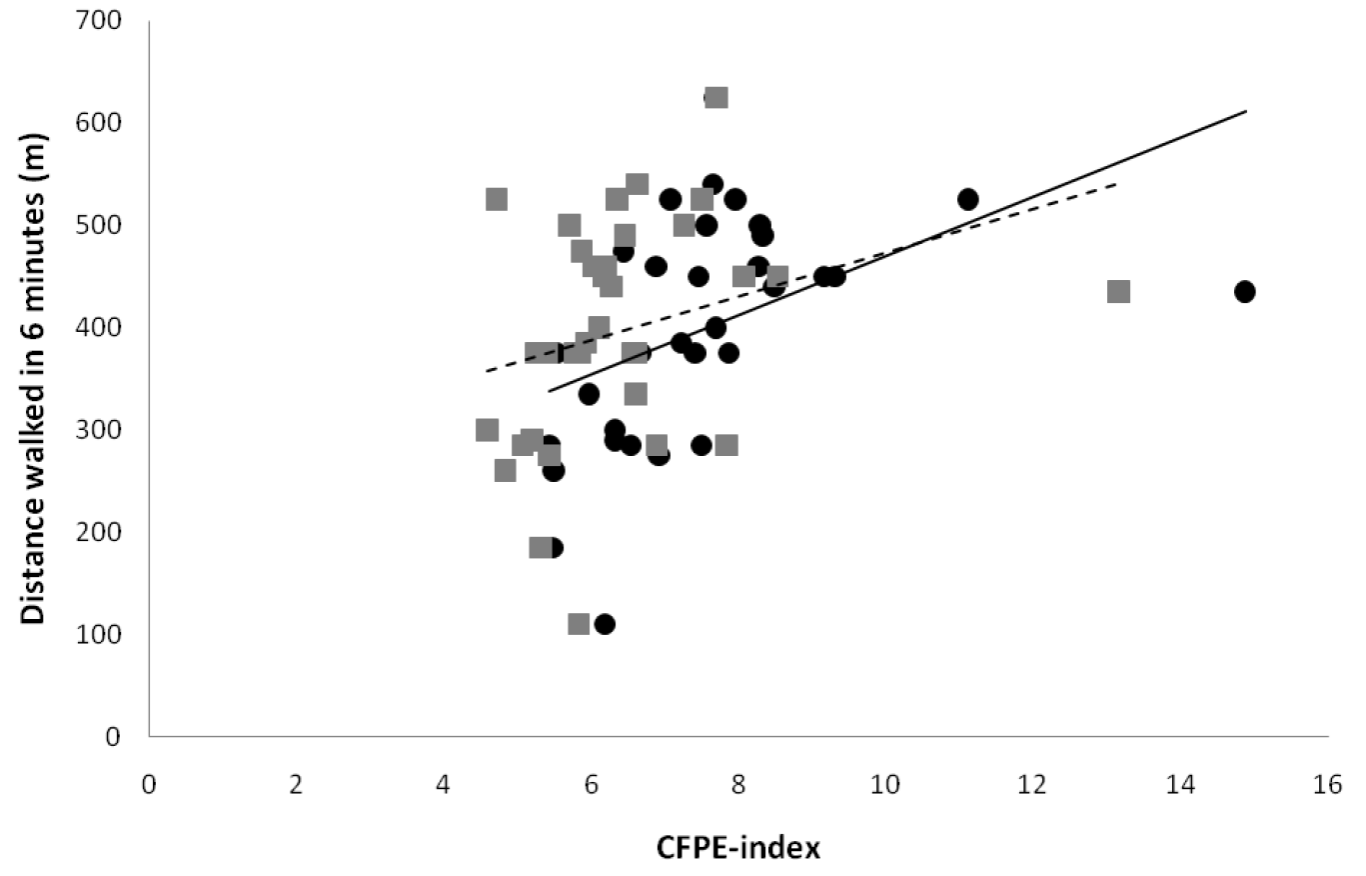
**Relationship between distance walked in six minutes and CFPE-index for type I and type IIa fibres; "black circle" = type I fibres (-- = regression line for CFPE-index for type I fibres, r = 0.67, p < 0.001), "grey square"= type IIa fibres (-- = regression line for CFPE-index for type IIa fibres, r = 0.40, p = 0.02); CFPE-index = quotient between individual capillary-to-fibre ratio and fibre perimeter**.

**Figure 3:**
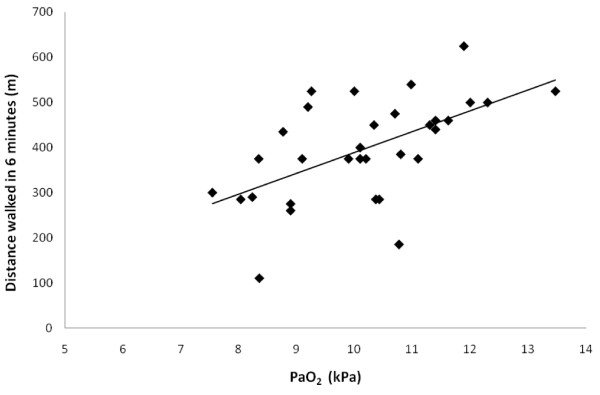
**Relationship between exercise capacity expressed as distance walked in six minutes and partial oxygen pressure (PaO_2_), r = 0.57, p < 0.001**.

A positive correlation was observed between exercise capacity, expressed as distance walked in six minutes, and CFPE-index for both type I fibres (r = 0.67, p < 0.001) and type IIa fibres (r = 0.40, p = 0.02) (Fig 2) indicating a parallel reduction in exercise capacity and muscle capillarization. Exercise capacity, expressed as distance walked in six minutes, was also found to correlate positively to PaO_2 _(r = 0.57, p < 0.001) (Fig 3).

## Discussion

Previous studies have suggested alterations in the capillary bed of skeletal muscle in COPD patients. Extending these findings the current study provides first evidence of a disturbed muscle-to-capillary interface expressed as CFPE-index in COPD. Additionally, we offer evidence for a positive correlation between the degree of muscle capillarization, degree of airflow obstruction and exercise capacity in COPD patients.

The presence of an adequate capillarization is essential for maintenance of adequate oxygen supply required for normal muscle function. Recently, we have demonstrated an increased proportion of type IIa fibres and a decreased proportion of type I fibres in the tibialis anterior muscle of patients with COPD [10]. This is in line with previous studies [3,7-9] and together with previous findings of a decrease in oxidative enzyme activities in COPD [6,7] our findings indicate the occurrence of a shift towards a more glycolytic profile in the limb muscle of COPD patients. Furthermore, the number of capillaries around a single muscle fibre (CAF) was decreased in patients with COPD compared to healthy subjects, indicating decreased muscle capillarization in COPD [13]. However, CAF in relation to fibre area (CAFA) did not differ between healthy subjects and patients with COPD [13]. Capillary parameters essentially determining the diffusion distance (capillary density or CAFA) may, thus, reflect the reduction in fibre area in COPD patients [9,10] while disturbed capillarization may still be undetected. The current study has investigated muscle to capillary interface in COPD patients as this parameter is involved in oxygen supply to the muscle and has been suggested to be a sensitive marker for changes in the capillary network of limb muscle [14-19]. Our results demonstrate a positive relationship between the degree of airflow obstruction and CFPE-index for both type I and type IIa fibres, indicating that muscle capillarization decreases with increased disease severity. Furthermore, the CFPE-index for type I fibres, but not for type IIa fibres, was significantly reduced in patients with moderate and severe COPD compared to healthy subjects. A larger capillary network is associated with oxidative type I fibres compared to glycolytic type IIa fibres, which may explain why alterations in CFPE-index are more evident in the type I fibres. Interestingly, we found no differences in LC/PF between COPD patients and healthy subjects. A main difference between CFPE-index and LC/PF is that the calculation of CFPE-index relies on the measurement of the capillary-to-fibre ratio (C:F_i_, i.e. the sum of the fractional contributions of all capillary contacts around the fibre). We suggest that the C:F_i _is the capillary variable mainly affected in COPD patients compared to healthy subjects i.e. each capillary is shared by more muscle fibres. The specific alterations of this variable may be due to the fact that it is sensitive to alterations in the two-dimensional capillary-fibre geometrical arrangement [19]. We speculate that the presence of hypoxia in COPD leads to decreased oxygen delivery to the muscle fibres which may lead to rearrangements of the capillary-fibre geometry. Still, the mechanisms behind these rearrangements are not known and further studies are needed to confirm these speculations. However, as the CFPE-index is considered a sensitive marker for changes in muscle-to-capillary interface [16], we conclude that the interface is disturbed in the tibialis anterior muscle of COPD patients. As it has previously been reported that muscle-to-capillary interface measured as capillary-to-fibre surface ratio is regulated as a function of the fibre mitochondrial volume per length of fibre [26] another plausible explanation to our findings may be a reduced mitochondrial volume of the muscle fibre. This explanation would be in line with a recent study showing a decrease in mitochondrial volume in patients with COPD [12]. However, further studies on the relationship between capillarization and mitochondrial volume density in COPD are needed to confirm this hypothesis.

Recently, we have demonstrated that decreased exercise capacity, as determined by the 6-min walking test, was strongly correlated with increased severity of COPD (p > 0.001) [10]. Here, we extend these findings and show that CFPE-index is also correlated to the degree of airflow obstruction as determined by spirometry and to exercise capacity determined by the 6-min walking test. In the context of motor unit recruitment during different muscular activities it is known that low intensity muscle activities mainly recruit low threshold slow, oxidative type I fibres, while the high threshold more glycolytic type IIa fibres are mainly recruited during high speed, high-force generating muscle activities. As walking is considered a low intensity activity the 6-min walking test recruits type I fibres to a larger extent than type II fibres [27]. As disturbance of the muscle-to-capillary interface is more pronounced for type I fibres the correlation between exercise capacity and CFPE-index is also stronger for type I fibres than for type IIa fibres. This strongly suggests a contributing role for decreased muscle capillarization and subsequently impaired oxygen delivery in the development of reduced exercise capacity in COPD. Indeed, a correlation between reduced muscle oxygen supply and reduction in exercise capacity in COPD has previously been suggested [11]. However, the finding of a strong correlation between exercise capacity and partial arterial pressure for oxygen indicates that other factors such as lung disease also are limiting factors for exercise capacity in COPD.

The mechanisms mediating decreased muscle capillarization in COPD are unknown. However, it is known that skeletal muscle adapts to physiological stimuli such as exercise and environmental factors such as hypoxia by changes in microvascularization [28,29]. As it has previously been suggested, one plausible explanation to the decreased capillarization may be the lower physical activity levels in COPD patients [30]. Another explanation to the findings in the present study may be the presence of hypoxia in COPD. This hypothesis is strengthened by a recent study where we have demonstrated an overexpression of the von Hippel-Lindau tumor suppressor protein (pVHL) in the tibialis anterior muscle of patients with COPD [13]. Increased pVHL may have an adverse effect on tissue capillarization as it impairs transduction of hypoxic-angiogenetic transcription factors including vascular endothelial growth factor (VEGF) [13,31]. Indeed, evidence for attenuation of VEGF gene expression during long term exposure to hypoxia has previously been demonstrated in skeletal muscle of rats [32]. Additionally, previous studies examining the effect of hypoxia on skeletal muscle have suggested that short term exposure to hypoxic conditions leads to an increase in capillaries/muscle fibre which is explained by a reduction in muscle fibre area and not by capillary neoformation [29,33,34]. During chronic exposure to hypoxia an actual reduction in muscle capillarity has been reported [29,35]. Taken together, these findings indicate that the presence of a hypoxic state may account for decreased skeletal muscle capillarization in COPD. However, further studies are needed to confirm this theory.

In conclusion, the present study provides evidence of a positive correlation between decreased muscle-to-capillary interface and increased disease severity in COPD. Furthermore, a positive correlation is demonstrated between decreased muscle-to-capillary interface and decreased exercise capacity in patients with COPD. Exercise is known to have a positive effect on muscle-to-capillary interface [16], which highlights the need to develop rehabilitation strategies to promote capillarization, improve oxygen delivery and consequently improve exercise capacity in COPD patients.

## Competing interests

The authors declare that they have no competing interests.

## Authors' contributions

GE carried out the exercise capacity test and the immunohistochemistry, participated in the design of the study, performed the statistical analysis and drafted the manuscript. SA-H participated in the design of the study as well as patient recruitment. KP-A performed the muscle biopsy sampling and participated in the design and coordination of the study. FK conceived the study and helped on the draft of the manuscript. All authors read and approved the final manuscript.
